# Electrooxidation Is a Promising Approach to Functionalization of Pyrazole-Type Compounds

**DOI:** 10.3390/molecules26164749

**Published:** 2021-08-05

**Authors:** Boris V. Lyalin, Vera L. Sigacheva, Anastasia S. Kudinova, Sergey V. Neverov, Vladimir A. Kokorekin, Vladimir A. Petrosyan

**Affiliations:** 1 N.D. Zelinsky Institute of Organic Chemistry, Russian Academy of Sciences, Leninsky Prosp. 47, 119991 Moscow, Russia; bv.lyalin@yandex.ru (B.V.L.); vsigaveva@yandex.ru (V.L.S.); ana_kudinova@mail.ru (A.S.K.); sergei.neverov@yandex.ru (S.V.N.); vladim.petros@yandex.ru (V.A.P.); 2 Institute of Pharmacy, Sechenov First Moscow State Medical University (Sechenov University), Trubetskaya Str. 8, Bldg. 2, 119991 Moscow, Russia; 3 All-Russian Research Institute of Phytopathology, Institute Str. 5, 143050 Bol’shiye Vyazemy, Russia

**Keywords:** pyrazoles, pyrazolo[1,5-a]pyrimidines, aromatic substitution, electrooxidation, C–H halogenation, C–H thiocyanation, N–N coupling, cyclic voltammetry

## Abstract

The review summarizes for the first time the poorly studied electrooxidative functionalization of pyrazole derivatives leading to the C–Cl, C–Br, C–I, C–S and N–N coupling products with applied properties. The introduction discusses some aspects of aromatic hydrogen substitution. Further, we mainly consider our works on effective synthesis of the corresponding halogeno, thiocyanato and azo compounds using cheap, affordable and environmentally promising electric currents.

## 1. Introduction

The functionalization of arenes is the key to their diversity, opening the way to practically useful substances. At the beginning of the 21st century, C‒H functionalization of arenes became a popular tool for the implementation of such processes [1], and the center for selective C‒H functionalization (mainly based on metal complex catalysis) was organized in the USA [2,3]. At the same time, a more attractive metal-free C‒H functionalization of arenes has existed for many years. Its development is discussed below, since the review is related to it.

**Scheme 1:**
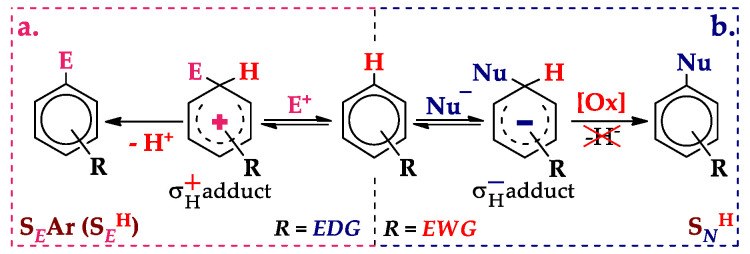
Electrophilic (**a**) and nucleophilic (**b**) aromatic hydrogen substitution.

The first to consider is the electrophilic aromatic hydrogen substitution (S_E_^H^) [4]. It proceeds via the σ_H_^+^ adduct formation and proton elimination leading to the target product (Scheme 1a). 

The nucleophilic aromatic hydrogen substitution (S_N_^H^, Scheme 1b) is problematic due to the difficulty of the hydride ion direct elimination from the σ_H_^−^ adduct. Nevertheless, in the mid-1970s, Chupakhin and Postovsky proposed an indirect way of obtaining the target product, the chemical oxidation of the σ_H_^−^ adduct [5]. To date, such processes have been extensively developed by Chupakhin and Charushin [6,7,8,9].

**Scheme 2:**
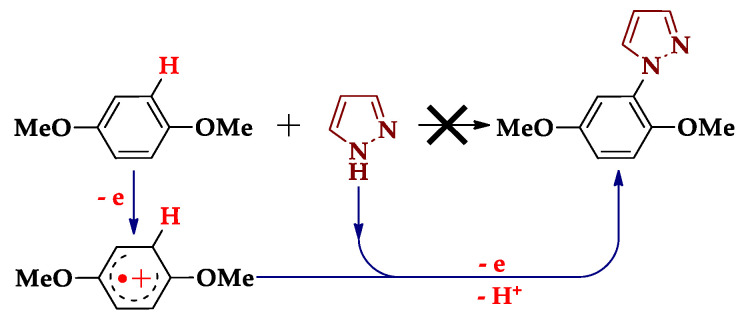
Electrooxidative *N*-arylation of pyrazole by 1,4-dimethoxybenzene.

This review is devoted to electrooxidative functionalization of pyrazoles. Why is it so attractive? The answer is given by Seebach [10], who showed that the interaction of two near-polar co-reactants is provided by the polarity inversion of one of them. This is the essence of electrochemistry, where the electrode transformation of the substrate is accompanied by its polarity inversion. For example, 1,4-dimethoxybenzene (DMB) and pyrazole (Scheme 2) are two non-interacting nucleophiles, but polarity inversion of DMB by electrooxidation leads to the electrophilic radical cation that reacts with pyrazole. In the electrochemical literature, this C‒H functionalization is called anodic substitution, which has been actively studied since the mid-1950s [11,12,13,14,15,16,17,18,19,20]. 

Note that chemical (electrophilic and nucleophilic) substitution and electrochemical (anodic) substitution in arenes have been being developed in parallel and independently of each other for a long time. In addition, the role of anodic substitution among the corresponding chemical processes was unconsidered. 

**Scheme 3:**
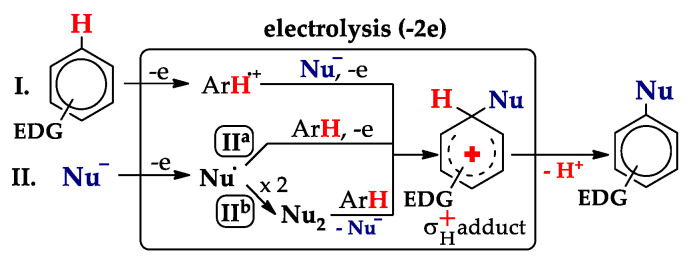
Main routes of S_N_^H^ An processes: **I** (E_p_^ox^_Ar_ < E_p_^ox^_Nu_^−^) or **II** (E_p_^ox^_Nu_^−^ < E_p_^ox^_Ar_).

Only recently we have introduced the concept of anodic substitution as an electrochemical aromatic substitution of hydrogen [21,22]. Such reactions were designated as S_N_^H^ An (An is anode), since the key stage is anodic oxidation of the substrate. For electron-rich arenes, the processes proceed along two main routes (Scheme 3), depending on the ease of oxidation of nucleophile vs. arene [21]. Route I (arene oxidizes easier than Nu^−^) proceeds via the interaction between Nu^−^ and the arene radical cation. Route II (Nu^−^ oxidizes easier than arene) proceeds via the formation of Nu^•^, which either interacts with the arenes homolytically (route II^a^) or forms a dimer that interacts with the arene as an electrophile (route II^b^).

**Scheme 4:**
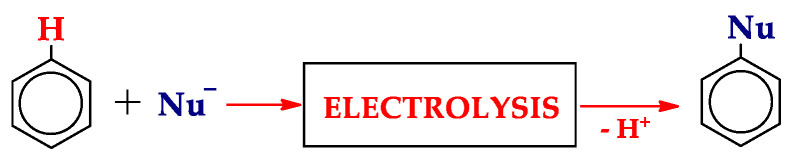
General scheme of the electrooxidative C‒H functionalization (C–H An).

In general, the multitude of S_N_^H^ (An) processes, where hydrogen is displaced with a nucleophile, are electrooxidative C‒H functionalizations (C–H An) and can be described by Scheme 4. Such strategy opens up the direct method of C–H functionalization of arenes with the C–C and C–Het coupling realization. The latter is especially shown in the examples of electrooxidative C–H halogenation and thiocyanation of pyrazole derivatives (Section 2 and Section 3). A special place is occupied by the N–N coupling of amino pyrazoles via the N–H functionalization (Section 4), since its patterns are somehow similar to those described above, but not sufficiently studied.

Such processes are attractive for green chemistry [23,24] because they use cheap, affordable and environmentally promising electric current instead of chemical oxidants (often toxic, unrecyclable and used in excess) and complex catalysts (sometimes expensive and toxic). In addition, the varying anode potential eliminates the difficulties of an empirical search for suitable chemical oxidants, while Pt, a frequently used electrode material [25], can be replaced by more attractive ones, e.g., glassy carbon, or ruthenium–titanium oxide (for more information on electrode materials see [25,26,27]). 

Since the review is devoted to the poorly studied electrochemical functionalization of pyrazole derivatives, Section 2, Section 3 and Section 4 mainly summarize the investigations of the review’s authors.

## 2. Electrooxidative C–H Halogenation of Pyrazole and Its Substituted Derivatives

Halogenated pyrazoles are widely used in organic synthesis; in particular, iodo and bromo pyrazoles are the key reagents in transition metal-catalyzed cross-coupling [28]. Moreover, they are important precursors of drugs, such as antihepatitis, anti-Alzheimer, antiparkinsonian and anti-schizophrenic drugs (chloro-pyrazoles) [29,30,31], antiglaucoma drugs (bromo-pyrazoles) [32] and antiatherosclerotic, antimalarial, anti-inflammatory and immunocorrective drugs (iodo-pyrazoles) [33,34,35,36,37,38]. At the same time, chloro- and iodo-pyrazoles are used for preparation of antidiabetic drugs [39,40], bromo- and iodo-pyrazoles—of anticancer [41,42,43,44] and antimicrobial [45,46] agents, and chloro- and bromo-pyrazoles—of agrochemicals [47,48,49,50,51].

The active use of halogeno-pyrazoles has stimulated interest in their efficient and ecologically attractive synthesis, including electrosynthesis (see Introduction). At the same time, the electrochemical halogenation of pyrazoles has practically not been studied before us, but it was preceded by chemical halogenation, the aspects of which are briefly given below.

### 2.1. Chemical Halogenation of Pyrazoles

**Scheme 5:**
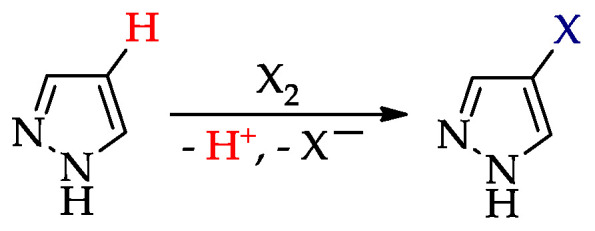
Chemical halogenation of pyrazoles (X = Cl, Br, I).

The processes (Scheme 5) are usually carried out by the interaction of pyrazoles and halogens (or halogenating reagents), and they occur first at position 4 and only then at other positions [28]. 

#### 2.1.1. Chlorination

The most common is the interaction of pyrazoles and Cl_2_. It was used to convert the pyrazole and its alkyl derivatives into the 4-chloro pyrazoles (yields 40–85%, at 0–40 °C, in CH_2_Cl_2_ or CCl_4_). Under severe conditions (at 80–100 °C, in AcOH) dichloropyrazoles and the chloro products of the alkyl groups were also formed [52,53]. At the same time, the corresponding 4-chloro derivatives were obtained by the reaction of 3,5-dimethyl-1*H*-pyrazole (and its *N*-substituted derivatives) with *N*-chlorosuccinimide (yield 95–98%, at 20–25 °C, in CCl_4_ or H_2_O) [54,55].

#### 2.1.2. Bromination

The reaction of alkyl-substituted pyrazoles and their carboxylic acids with Br_2_ (at 20–25 °C) proceeded in yields of 75–96% in non-aqueous media (CH_2_Cl_2_, CHCl_3_ or CCl_4_) [45,56,57,58], or 50–75% in water [59,60]. The NaOH additives (which binds with HBr formed) allows bromination of low-reactive pyrazole-3-carboxylic acid in the yield of 90% [61]. A number of 4-bromopyrazoles were also obtained by the reaction of 3,5-dimethyl-1*H*-pyrazole (and its *N*-substituted derivatives) with *N*-bromosuccinimide (yields 90–99%, at 20–25 °C, in CCl_4_ or H_2_O) [55].

#### 2.1.3. Iodination

Good results in the iodination of pyrazoles with donor substituents were obtained using the I_2_–NaI–K_2_CO_3_ system (yields 75–90% at 20–25 °C in aq. EtOH) [62,63,64]. A solution of N-iodosuccinimide in acidic media (50% aq. H_2_SO_4_, CF_3_SO_3_H, CF_3_COOH, AcOH) was also efficient for the iodination [44,62,65]. Finally, the iodination of practically any pyrazoles proceeded efficiently and without toxic waste using the I_2_–HIO_3_ system in AcOH–CCl_4_ [66,67].

In general, the above methods are quite effective, but not ecologically attractive enough due to the frequent use of halogens in their pure form or waste of other halogenating agents (e.g., succinimide). Such problems can be solved using electrochemical methods—C–H An halogenation. 

### 2.2. C-H An Halogenation of Pyrazoles

**Scheme 6:**
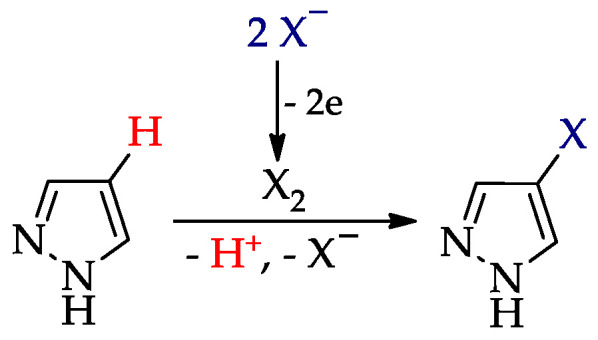
C–H An halogenation of pyrazoles (X = Cl, Br, I).

The halogenation (Scheme 6) usually proceeds [26] via the electrogeneration of a halogen followed by its interaction with pyrazole (cf. Scheme 3, route II^b^).

Such processes are mainly carried out under mild conditions in an anodic compartment of a divided cell on a Pt-anode under galvanostatic electrolysis with alkali metal halides in H_2_O or in H_2_O–CHCl_3_. A series of *N–H* and *N–Alk* pyrazoles, including those with donor (acceptor) substituents, were objects of study (Table 1, Table 2 and Table 3).

#### 2.2.1. Chlorination

**Scheme 7:**
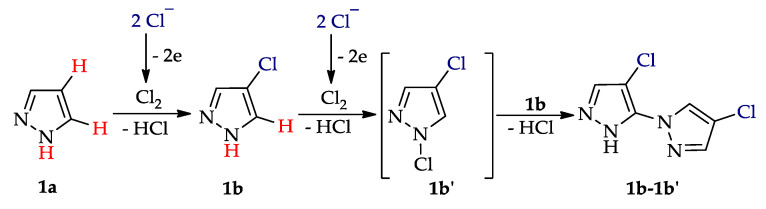
C‒H An chlorination of pyrazole **1a**.

C–H An chlorination of pyrazole **1a** (Table 1, entry 1) led to 4-chloropyrazole **1b** (yield 46%) and to by-product **1b′** (yield 8%) in H_2_O–CHCl_3_ at the theoretical amount of electricity passed (Q/Q_t_ = 1). Apparently, the product **1b** undergoes chlorination to 1,4-dichloropyrazole **1b′** (Scheme 7), followed by C–N dehydrogenative cross-coupling to by-product **1b-1b′**. 

The need for CHCl_3_ (as an extractant of target product) should be noted, since its absence decreased the yield of product **1b** to 34% and increased the yield of by-product **1b-1b′** to 15%. Pyrazoles **2a**–**7a** (entries 2–7), gave the monochlorinated products **2b–7b** with different yields (8–71%) depending on the position of Me groups. Di- and trichloroproducts were obtained in entries 5 and 6.

Pyrazoles with acceptor groups (NO_2_ or COOH) were chlorinated without CHCl_3_ additives: the yields of the target products **8b**–**14b** were 41–93% (entries 8–14). Only pyrazole **14a**, containing both NO_2_ and COOH groups, was the least reactive. Therefore, the electrochemical method for the synthesis of 4-chloropyrazolcarboxylic acids [68] is noticeably superior to the corresponding chemical one [69].

#### 2.2.2. Bromination

Compared with chlorination, the C‒H An bromination (Table 2) proceeded more effectively for pyrazole and its methyl derivatives (yields of products **1c**–**6c** 55–94%). In some cases, dibromo by-products (entries 2 and 5), low yield (entry 9), or the absence of any reactions (entries 7 and 14) were observed. 

#### 2.2.3. Iodination 

C‒H An iodination by weakly electrophilic I_2_ (Table 3) was generally less effective than bromination [73]. Traces of the target products or no reaction were observed in half of the cases (entries 2, 7–9, 11–13). In other cases, the yields were 35–86% (for pyrazole and its methyl derivatives in entries 1, 3–6) and 30–40% (for nitro- and carboxypyrazoles in entries 10 and 14).

**Scheme 8:**
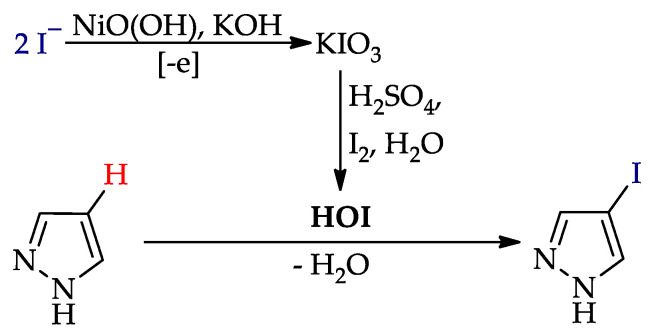
C–H iodination of pyrazoles via HOI.

A much more effective iodinating agent was HOI, which can be obtained by the reaction of KIO_3_ with KI (or I_2_) and H_2_SO_4_ [75,76,77,78,79]. The original process [74] includes the electrogeneration of KIO_3_ (on the NiO(OH) anode [80]), followed by the interaction of HOI generated in situ with the pyrazole (Scheme 8). As a result, the yields of target products increased to 74–93% (entries 1, 2, 4, 8, 10–12, 13). 

### 2.3. The Mechanistic Aspects of C–H (An) Halogenation of Pyrazoles

**Scheme 9:**
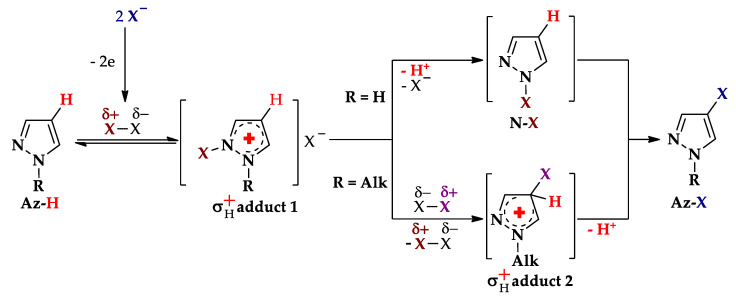
C–H An halogenation of pyrazoles (possible mechanisms, X = Cl, Br, I).

Since I^−^, Br^−^, and Cl^−^ are commonly oxidized at lower anodic potentials than the studied pyrazoles, the process proceeds via the electrooxidation of Hal^−^ to Hal_2_ followed by interaction of the latter with arenes (see Scheme 3, route II^b^ and Scheme 6). The possible mechanism [26,71,79] (Scheme 9) includes the initial attack of the halogen on the N^2^ of **Az–H** with the formation of **σ_H_^+^ adduct 1**. The latter, depending on the **R**, gives either **N–X** intermediate (R = H) or **σ_H_^+^ adduct 2** (R = Alk). Therefore, the target **Az–X** is formed either due to N–C rearrangement of **N–X** derivative or due to the deprotonation of **σ_H_^+^ adduct 2**. 

**Scheme 10:**
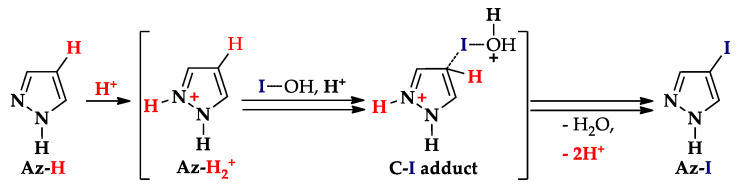
Iodination of highly basic pyrazoles by HOI.

Iodination by HOI proceeds similarly, but for highly basic N-unsubstituted pyrazole and its alkyl derivatives it most likely occurs (Scheme 10) via **C–I adduct** (the result of protonation of N^2^ and HOI attack on C^4^) [74,75,77,78,79].

**Scheme 11:**
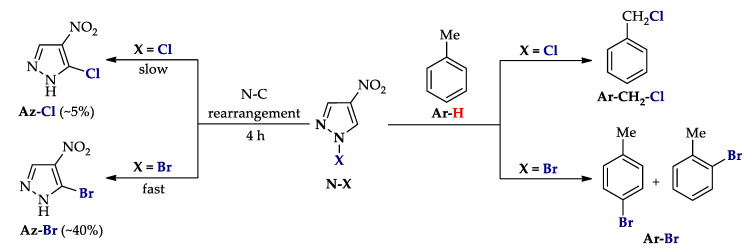
Different behavior of N–Cl and N–Br intermediates (unpublished control experiments).

Additional control experiments showed different properties of N–Cl and N–Br intermediates (Scheme 11). Therefore, the N–C rearrangement of the N–Cl derivative is significantly lower than that for the N–Br (cf. stages **N–X**→**Az–Cl** and **N–X**→**Az–Br**). At the same time, for the N–Cl bond, homolytic cleavage is observed, while for N–Br it is heterolytic (cf. stages **N–X→Ar–CH_2_-Cl** and **N–X→Ar–Br**).

The above data not only reveal the essence of pyrazoles C‒H An halogenation, but also explain the difference in its efficiency (e.g., the anomalously less efficient chlorination of N-unsubstituted pyrazoles compared to bromination (cf. entries 1, 3 and 4, Table 1 and Table 2), and the formation of by-products (e.g., entries 1 and 6, Table 1).

Therefore, this Section describes the basic patterns of C–H An halogenation, and the efficient (up to 94% yield) gram-scale synthesis of a series of chloro-, bromo- and iodo-pyrazoles in aqueous or aqueous-organic media. The following Section reflects the main points on the related C‒H An thiocyanation of pyrazole derivatives.

## 3. Electrooxidative C–H Thiocyanation of 5-Aminopyrazoles and Pyrazolo [1,5-a]pyrimidines

Thiocyanation of the C–H bond of arenes is an effective tool for C–S coupling [81,82,83,84]. The resulting aryl thiocyanates are valuable precursors of sulfur and nitrogen-containing compounds (thiols [85], (di)sulfides [86,87], dithiocarbamates [88], thiazoles [89], tetrazoles [90]), and are highly bioactive compounds (antifungal [91], antitumor [92], antiparasitic [93]). Recently synthesized thiocyanates of pyrazole derivatives also have sufficient antifungal [94] and antitumor [95] activity. 

**Scheme 12:**
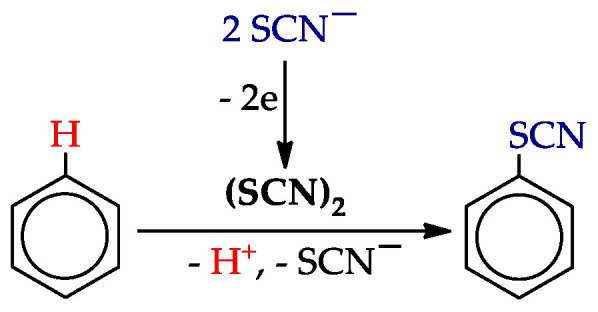
C–H thiocyanation of arenes via the thiocyanogen.

One of the key intermediates of C‒H thiocyanation of arenes is the well-known [96,97] pseudohalogene thiocyanogen (SCN)_2_. It is usually obtained in situ by chemical or electrochemical oxidation of the thiocyanate ion (Scheme 12).

**Scheme 13:**
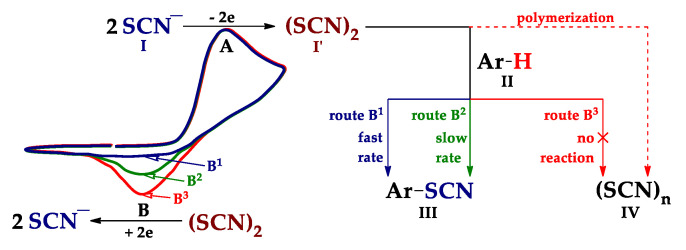
C–H An thiocyanation of arenes (**II**) via the thiocyanogen (**I′**) and voltammetric test of the process efficiency.

The chemical approach has been actively developed over the past 10–15 years, but it is often associated with the use of an excess of unrecyclable oxidants, which can sometimes be toxic, scalding or poorly available (e.g., Br_2_ [98], I_2_ [99], DEAD [100], HIO_3_ [101], H_5_IO_6_ [102], I_2_O_5_ [103], H_2_O_2_ [102,104,105,106,107], K_2_S_2_O_8_ [108,109], CAN [110], Mn(OAc)_3_ [111], *p*-TSA [112], NCS [113], NBS [100], NIS [114], NTS [115], DDQ [116,117]). The electrochemical approach (see [22], Scheme 3, route II^b^, and Scheme 13) is devoid of such disadvantages, but it is poorly studied in general [118,119,120]. For pyrazole derivatives, C–H An thiocyanation is studied for the first time in a series of works [22,121,122,123,124,125,126], which are reflected in this Section. 

### 3.1. C–H An Thiocyanation: General Patterns and Approaches

According to the above and developed [22,118,119,120,121,122,123,124,125,126] concepts, C–H An thiocyanation occurs during the anodic oxidation of the thiocyanate ion in the presence of arene, as a rule, via the thiocyanogen (Scheme 13, step **I→I′**). The latter either interacts with arene (step **I′ + II→III**), or gives polythiocyanogen [127] (step **I′→IV**).

These processes were investigated by cyclic voltammetry (CV) [22,123,125,126]. Scheme 13 shows a typical CV curve of SCN^−^. Peak **A** corresponds to the oxidation of thiocyanate ion **I** to thiocyanogen **I′**, which is detected on the reverse scan by its reduction peak **B** (**B^3^**). If after the addition of arene **II,** peak **B** disappears (cf. peaks **B^1^** and **B^3^**) or decreases (cf. peaks **B^2^** and **B^3^**), then Ar–H **II** interacts with (SCN)_2_, respectively, via the route **B^1^** or **B^2^** to form the target Ar–SCN **III**. If the peak **B** does not change, then Ar–H **II** does not react with (SCN)_2_ (see peak B^3^ and route B^3^). In this case the main reaction product is polythiocyanogen **IV**.

**Scheme 14:**
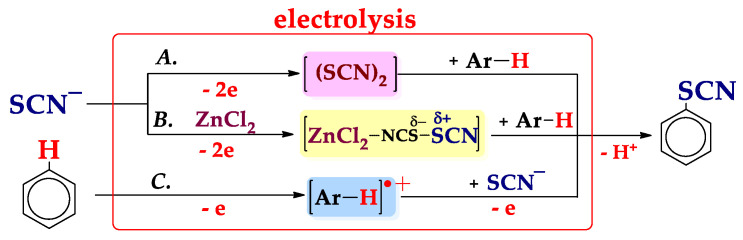
Approaches and possible mechanisms of C–H An thiocyanation.

Further, we proposed [122,123] the original system of approaches to the C–H An thiocyanation of arenes (Scheme 14) depending on the reactivity of arenes with respect to (SCN)_2_: via the generation (SCN)_2_ at the oxidation potential (E_p_^ox^) of thiocyanate ion (approach A, cf. Scheme 3, route II^b^, and Scheme 13), via electrogeneration (SCN)_2_ in the presence of ZnCl_2_ activating additives (approach B) or via the generation of a highly reactive radical cation at E_p_^ox^ of arene (approach C, cf. Scheme 3, route I). 

Approach A is used for arenes that react with (SCN)_2_ (Scheme 13, routes B^1^ and B^2^), whereas approaches B and C are used for arenes that do not interact with non-activated (SCN)_2_. 

These patterns and approaches are considered below on the examples of C–H An thiocyanation of the practically useful [128,129,130] derivatives of 5-aminopyrazole and pyrazolo[1,5-a]pyrimidine and the original electrosynthesized 1-(hetero)arylpyrazoles [124,131].

### 3.2. C–H An Thiocyanation of Pyrazole Derivatives

The studies included a preliminary CV test in addition to electrosynthesis. The initial pyrazoles **1e–15e** and their thiocyanation products **1f–15f** are presented in Table 4 and Table 5.

#### 3.2.1. CV studies and the Choice of Optimal Approach

**Figure 1:**
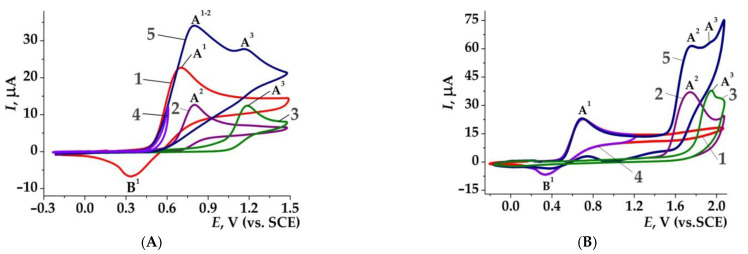
CV curves on Pt working electrode in 0.1M NaClO_4_ in MeCN, ν = 0.10 V·s^−1^. (**A**) NH_4_SCN (0.002M)—1; 3-methyl-1*H*-pyrazol-5-amine **1e** (0.002M)—2; 3-methyl-4-thiocyanato-1*H*-pyrazol-5-amine **1f** (0.002M)—3; mixture NH_4_SCN/azole **1e** (1:1) with the reverse scan from 0.60 V—4; the same on the reverse scan from 1.45V—5; (**B**) NH_4_SCN (0.002M)—1; 2-methyl-5-thiophen-2-yl-7-(trifluoromethyl)pyrazolo[1,5-a]pyrimidine **10e**—2; 2-methyl-3-thiocyanato-5-thiophen-2-yl-7-(trifluoromethyl)pyrazolo[1,5-a]pyrimidine **10f**—3; mixture NH_4_SCN/ azole **10e** (1:1) on the reverse scan from 1.20 V—4; the same on the reverse scan from 2.10 V—5.

Figure 1 shows CV curves of NH_4_SCN and its mixtures with 3-methyl-1*H*-pyrazol-5-amine (**1e**), 2-methyl-5-thiophen-2-yl-7-(trifluoromethyl) pyrazolo[1,5-a]pyrimidine (**10e**), as well as curves of individual compounds and their thiocyanato products (**1e**,**10e,1f**,**10f**) [125,126]. The CV of NH_4_SCN (curve 1, Figure 1A,B) has the anodic peak A^1^ (E_p_^ox^ = 0.70 V) of the thiocyanate ion and a cathodic peak B^1^ (E_p_^red^ = 0.34 V) of the thiocyanogen. The peak B^1^ disappeared after the addition of pyrazole **1e** and did not change after the addition of pyrazole **10e** (cf. corresponding curves 1 and 4). This clearly shows that pyrazole **1e** reacts rapidly with (SCN)_2_ (see Scheme 13, route B^1^) and approach A (Scheme 14) is suitable for its thiocyanation. From the other side, the pyrazole **10e** does not react with (SCN)_2_ and approaches B and C may be suitable for its thiocyanation. Note also that on full scans, peaks A^3^ of thiocyanates **1f**,**10f** were observed, in addition to the peaks **A^2^** of pyrazoles **1e**,**10e** (see curve 5, Figure 1A,B).

#### 3.2.2. Electrosynthesis

Electrolyses were carried out in 0.1M solution of NaClO_4_ in MeCN (MeCN–H_2_O) in undivided or divided cells (UC or DC) in controlled-potential or galvanostatic mode (CPE or GE), passing a theoretical or excess amounts of electricity (Q/Q_t_ = 1–3). Pt or glassy carbon (GC) electrodes were used.

The amino compounds **1e–6e** gave thiocyanates **1f–6f** with yields 64–89% (under CPE at E_p_^ox^_SCN_^−^) and 57–71% (under GE) at Q/Q_t_ = 1 (Table 4, entries 1–6) when implementing approach A (see Scheme 14). 

From the less reactive pyrazolo[1,5-a]pyrimidines **7e**–**9e**, products **7f**–**9f** were obtained with yields 66–85% at Q/Q_t_ = 2 (entries 7–9). The electrode material affected things differently: the yield of thiocyanate **2a** (entry 1) was 83% (GC) and 72% (Pt), while the yield of thiocyanate **2g** (entry 7) was 64% (GC) and 89% (Pt). Most of the processes (entries 3–9) were successfully carried out in an undivided cell. The possibility of scaling the process was also shown (entries 1, 2 and 7).

It was noted [121,122,123,125] that approach A is not suitable for the thiocyanation of hardly oxidizable (E_p_^ox^ > 1.70 V) pyrazoles **10e**–**15e** with acceptor substituents (Table 5) and leads to trace amounts of target thiocyanates **10f**–**15f** and polythiocyanogen (see Scheme 13, route B^3^). In this case, the process proceeds quite efficiently with an increase in the reactivity of the thiocyanogen (Scheme 14, approach B) or the initial pyrazole (approach C). As a result, CPE in the presence of ZnCl_2_ activating additives (approach B) allowed us to obtain products **10f–15f** with yields 69–81% [121,123], while metal-free CPE at E_p_^ox^_AzH_ with Q/Q_t_ = 3 (approach C) also led to the products **10f**–**15f** with smaller yields of 47–65% [122,123].

Note that the possibility of approach C realization can also be tested by CV: a decrease in the peak B^1^ is observed on the full scan (cf. curves 5 and 1, Figure 1B), which corresponds to the interaction of the thiocyanate ion and the pyrazole cation radical via the ECE mechanism [122,123,132] (see Scheme 14, approach C, and Scheme 3, route I).

**Scheme 15:**
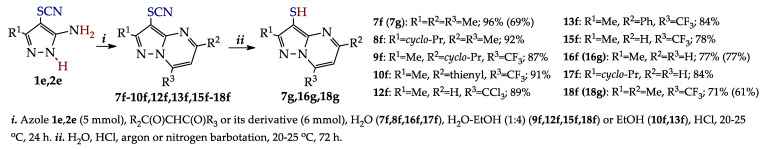
Synthesis of 3-thiocyanatopyrazolo[1,5-a]pyrimidines and pyrazolo[1,5-a]pyrimidine-3-thiols.

In addition to approaches A–C, an equally effective approach was developed [41] based on the HCl-catalyzed condensation of previously obtained 4-thiocyanatopyrazoles **1e**, **2e** (see entries 1 and 2, Table 4) with 1,3-dicarbonyl compounds or their derivatives (Scheme 15). As a result, 3-thiocyanatopyrazolo[1,5-a]pyrimidines both without substituents and with donor (acceptor) substituents in the pyrimidine ring were obtained with yields of 77–96% [126].

Developing this direction, the opportunity of transformation of the SCN group into the SH group [94,123] was shown, which opens the way to thiols as promising nucleophiles for C–H functionalization (e.g., see [133,134,135]). Hydrolysis with HCl was the most effective (yields of thiols **7g**, **16g**, **18g** were 61–77%), while the use of chemical reductants or strong acids (LiAlH_4_, NaBH_4_, Zn in AcOH, HClO_4_, H_2_SO_4_) was ineffective.

Thus, a series of thiocyanates of substituted pyrazoles and pyrazolo[1,5-a]pyrimidines were obtained on the basis of electrolysis of the “thiocyanate ion/pyrazole” mixture.

**Scheme 16:**
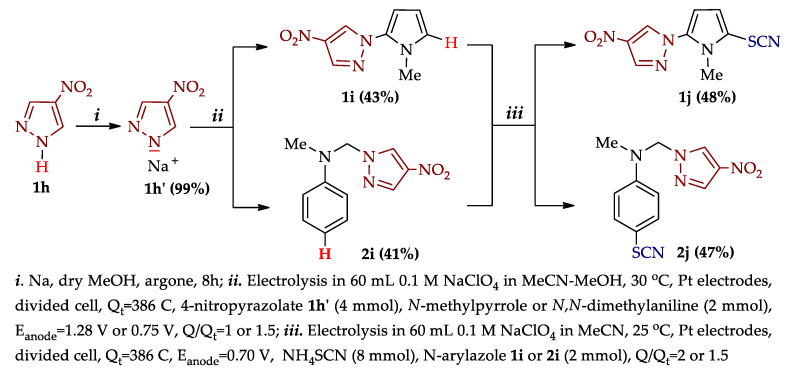
N–H An arylation of 4-nitropyrazole followed by C–H An thiocyanation of resulting N-arylazoles.

In addition, during the development of research on the electrosynthesis of aryl thiocyanates [22] and N-arylpyrazoles [131,136,137,138] we showed [124] the possibility of synthesizing new molecules with pyrazole and thiocyanate fragments (Scheme 16) as promising hybrid polyfunctional [139] structures.

The N–H arylation of pyrazole **1h** was carried out by activating its N–H bond (i) followed by the introduction of electrolysis with N-methylpyrrole or N,N-dimethylaniline (ii). In the latter case, the reaction proceeded selectively at the Me group without affecting the aromatic ring. Subsequent C–H An thiocyanation (iii) of the isolated N-arylazoles **1i**, **2i** led to the target products **1j**, **2j**.

### 3.3. Antifungal and Antibacterial Activity of Thiocyanated Pyrazole Derivatives

Tests for antifungal (*C. albicans*, *A. niger*) and antibacterial (*S. aureus*, *E. coli*) activity [91,94,123,124] showed that thiocyanate-pyrazoles are more active against fungi than bacteria. The greatest activity is observed against *A. niger* at thiocyanate **7f** [94] and thiocyanatoazolylaniline **2j** [124], whose minimum inhibitory concentration (MIC) is 0.24–0.48 µg/mL (it is superior to the antifungal drugs amphotericin B and fluconazole and is comparable to itraconazole).

**Figure 2:**
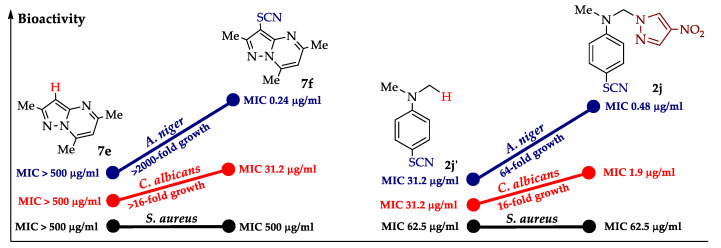
Effect of thiocyanate and pyrazole fragments on antifungal and antibacterial activity.

The contribution of thiocyanate and pyrazole fragments to antifungal and antibacterial activity was clearly shown in the individual examples (Figure 2). Thus, the activity of compound **1j** increased more than 2000-fold for *A. niger* and more than 16-fold for *C. albicans* after the introduction of the SCN group. The presence of 4-nitropyrazole in 4-thiocyanatoaniline **2j’** provided a selective increase in antifungal activity by a factor of 16–64, while *N*-arylazole **2i** was inactive in all cases.

Therefore, this Section is devoted to the efficient C–H An thiocyanation of various pyrazole derivatives (in some cases, their N–H An arylation), leading to pharmacologically active target mono- and polyfunctional products. The next Section is devoted to the N–H functionalization of amino pyrazoles followed by their N–N coupling and obtaining azopyrazoles.

## 4. (Electro)oxidative N–N Coupling of Aminopyrazoles

**Scheme 17:**
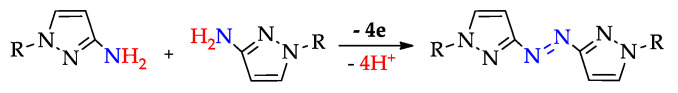
Synthesis of azopyrazoles by (electro)oxidative N–N coupling of aminopyrazoles.

Azoarenes are widely used in practice: from dyes and pharmaceuticals [140,141,142] to reagents in syntheses [143,144] and energy-rich materials [145,146]. One of the most popular methods for the synthesis of azoarenes is the oxidation of corresponding amines (Scheme 17), predominantly by chemical oxidants (BaMnO_4_ [147], Pb(OAc)_4_ [148], HgO [149], K_2_FeO_4_ [150], TCICA [151], t-BuOI [152]) or oxidation systems (CuBr-pyridine-O_2_ [153], I_2_-t-BuOOH [154], t-BuOCl-NaI [155]). The synthesis of polyfunctional azopyrazoles by silver catalyzed cascade conversion of diazo compounds [156] is also of interest.

At the same time, a more promising electrochemical approach is poorly studied. In particular, electrosyntheses of azobenzene on the Pt anode [157,158] or N,N’-bis(morpholino)diazene on the NiO(OH) anode [159] are described. Note that NiO(OH) is one of the popular electrogenerated redox mediators [80,159]. The use of such redox-mediators is a trend in modern electroorganic chemistry [18], since it allows the processes to be carried out under milder conditions, increasing their efficiency and selectivity. This Section describes the original approaches to the synthesis of azopyrazoles using electrogenerated redox mediators NiO(OH) [160,161,162] and Br_2_ [163], or electrogenerated hypohalites as oxidants [164,165].

### 4.1. (Electro)oxidative N-N Coupling of Aminopyrazoles: Approaches and General Patterns

**Scheme 18:**
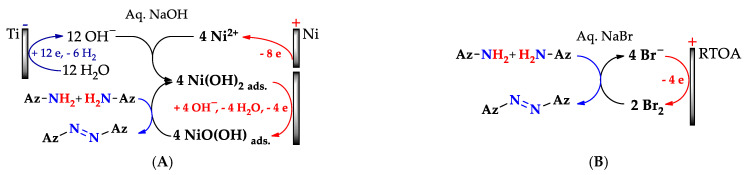
One-stage redox-mediated N–N coupling of aminopyrazoles using electrogenerated NiO(OH) (**Approach A**) and Br_2_ (**Approach B**).

One-stage Approach A (Scheme 18) is carried out in alkaline medium via the anodic dissolution of the Ni and the formation of adsorbed Ni(OH)_2_, followed by its anodic oxidation to adsorbed NiO(OH). It oxidizes aminopyrazoles (**Az–NH_2_**) to azopyrazoles (**Az–N = N–Az**) and forms Ni(OH)_2_, after which the cycle repeats [80,161]. In Approach B, the metal-free oxidant is Br_2_ [164], which is effectively electro(re)generated on the ruthenium–titanium oxide anode (RTOA).

**Figure 3:**
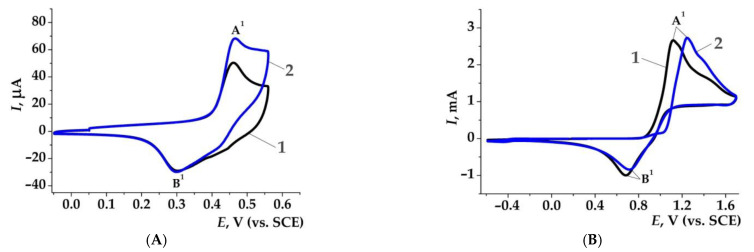
CV curves, ν = 0.10 V·s^−1^. (**A**) On Ni working electrode in 0.2M aq. NaOH: electrogenerated Ni(OH)_2_—1; after addition of 1-methyl-1*H*-pyrazol-3-amine **1k** (0.002M)—2; (**B**) on Pt working electrode in 1M aq. NaNO_3_: NaBr (0.3M)—1; after addition of 1-methyl-1*H*-pyrazol-3-amine **1k** (0.002M)—2.

In addition, special voltammetric tests showed that an increase in the Ni(OH)_2_ peak (Figure 3, A, peak A^1^, E_p_^ox^ = 0.46 V) or a decrease in the Br_2_ peak (Figure 3B, peak B^2^, E_p_^red^ = 0.69 V) after the adding of aminopyrazole is proportional to the process efficiency [162,164].

**Scheme 19:**
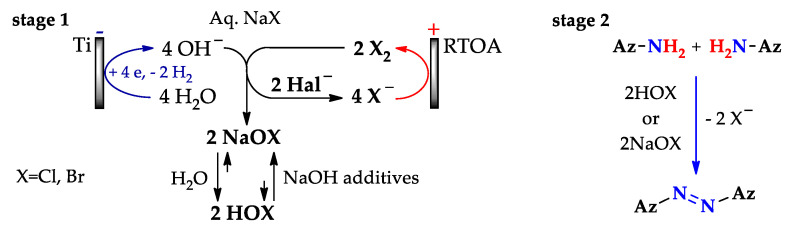
Two-stage N–N coupling of aminopyrazoles using electrogenerated HOCl (HOBr) or NaOCl (NaOBr) (Approaches C, D, C’, D’, respectively).

Two-stage approaches (Scheme 19) include preliminary electrogeneration of hypogalites followed by addition of aminopyrazoles [164,165]. Note, that hypohalites exist in equilibrium forms: predominantly HOCl (HOBr) in a neutral medium, and predominantly NaOCl (NaOBr) after adding NaOH (approaches C, D, C’, D’, respectively).

**Scheme 20:**
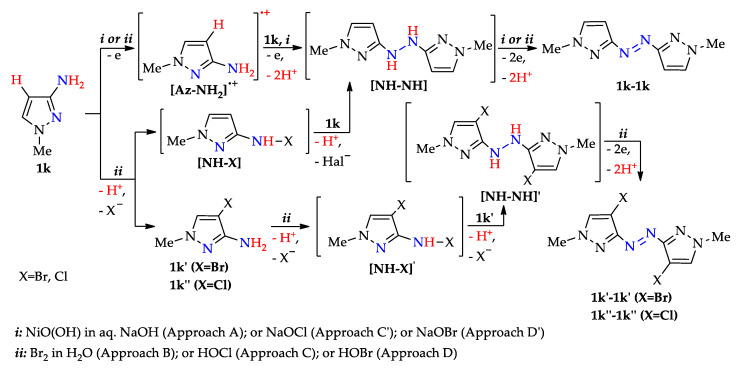
N–N coupling of aminopyrazoles (possible mechanisms).

According to the data [80,152,154,155,159,163,164,165], the possible mechanisms (Scheme 20) involve the oxidation of 1-methyl-1*H*-pyrazol-3-amine **1k** (step **1k→[Az–NH_2_]^·+^**) or its N–H halogenation (step **1k→[NH–X]**) followed by N–H amination to hydrazopyrazole **[NH–NH]** and its oxidation to the target azopyrazole **1k–1k** (see also Scheme 3 and Scheme 9).

When Br_2_ and HOX are used, C–H halogenation of aminopyrazole **1k** also occurs (step **1k**→**1k′**(**1k″**), see Scheme 9), followed by the formation of halogenated azopyrazole (steps **1k′→[NH–X]’→[NH–NH]’→1k′–1k′**(**1k″–1k″**)). These patterns are consistent with the experimental results below.

### 4.2. Synthesis of Azopyrazoles

Approach A is most versatile and allows us to obtain target azopyrazoles with yields of 52–88% (Table 6, entries 1–6, 8–11). On the contrary, approaches B, C and D are more suitable for 4-substituted pyrazoles (entries 2, 6 and 7) or pyrazole with acceptor (CF_3_) group (entry 12), where the yields of the corresponding azo products were 62–93%.

Nevertheless, approaches C’ and D’ (entries 1 and 4) allow to obtain rather selectively azopyrazoles **1k**–**1k** and **2k**–**2k** (yields 72–75%), and approaches C and D (entry 8) open the way to azohalogenopyrazoles **6k″**–**6k″** and **6k″**–**6k″** (yields 79–80%).

Moreover, the approach A is useful for previously unexplored chemical and electrochemical N–N cross-coupling of aminopyrazoles [162] (Table 7), and yields of the target azo compounds **1k**–**2k** and **4k**–**5k** were 48–50%. Such results create prospects for obtaining useful multifunctional azo compounds [152,156].

## 5. Conclusions

This review is the first step in summarizing the data on promising, but poorly studied electrooxidative functionalization of C‒H and N‒H bonds in pyrazole derivatives. It paves the way for the efficient synthesis of C‒Cl, C‒Br, C‒I, C‒S and N‒N coupling products using cheap, affordable and environmentally promising electric currents.

Additional advantages are the predominantly galvanostatic electrolysis mode and the reusability of commercially available electrodes, salts and solvents, as well as the gram-scalability of the processes. In half of the cases, a simple isolation of pure target products without chromatography is also possible. Moreover, the key regularities of the corresponding processes are considered, including the dependence of the efficiency of functionalization of pyrazoles on their structure and oxidation potential. An increasingly important role is played by cyclic voltammetry, which makes it possible both to study mechanisms and to predict the efficiency of synthesis.

All this makes the electrooxidative functionalization of pyrazole-type compounds very viable for further application and development.

## Figures and Tables

**Table 1:** C‒H An chlorination of pyrazoles (Az‒H) ^1^.

Entry	Az‒H, Az‒Cl and other Products (Yield, %) [70,71]				Entry	Az‒H, Az‒Cl (Yield, %) [68,70,71]	
**1**	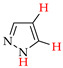 **1a**	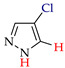 **1b** (46 ^2^)		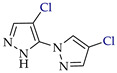 **1b-1b′** (8 ^2^)	**8**	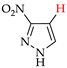 **8a**	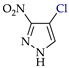 **8b** (41 ^2^)
**2**	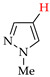 **2a**		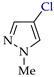 **2b** (71 ^3^)		**9**	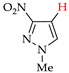 **9a**	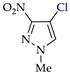 **9b** (64 ^3^)
**3**	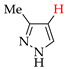 **3a**		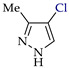 **3b** (34 ^2^)		**10**	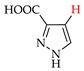 **10a**	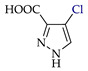 **10b** (92 ^3^)
**4**	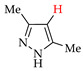 **4a**		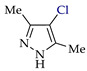 **4b** (70 ^3^)		**11**	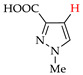 **11a**	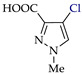 **11b** (69 ^3^)
**5**	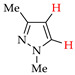 **5a**	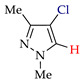 **5b** (15 ^2^)		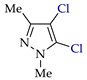 **5b’** (35 ^2^)	**12**	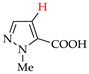 **12a**	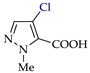 **12b** (93 ^3^)
**6**	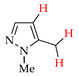 **6a**	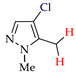 **6b** (47 ^2^)	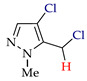 **6b’** (13 ^2^)	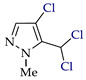 **6b’’** (4)^2^	**13**	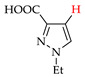 **13a**	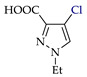 **13b** (84 ^3^)
**7**	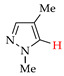 **7a**		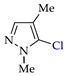 **7b** (8 ^2^)		**14**	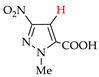 **14a**	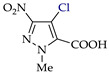 **14b** (4 ^2^)

^1^ Electrolysis in 100 mL of 4M solution of NaCl in H_2_O–CHCl_3_ (entries 1–7), H_2_O (entries 8–14), 15 °C, pyrazole (12.5–50 mmol), divided cell, Pt anode, Cu cathode, galvanostatic electrolysis (*j*_anode_ = 100 mA·cm^−2^), Q_t_ = 2412–9650 C, Q/Q_t_ = 1–2; ^2^ the yield was calculated from the ^1^H NMR spectroscopic data for the isolated mixture of products with unreacted pyrazoles; ^3^ the yield was determined for the isolated product.

**Table 2:** C‒H An bromination of pyrazoles (Az‒H) ^1^.

Entry	Az‒H, Az‒Br and Other Products (Yield, %) [71,72]				Entry	Az‒H, Az‒Br (Yield, %) [71,72]	
**1**	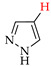 **1a**		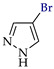 **1c** (70 ^2^)		**8**	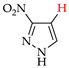 **8a**	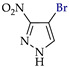 **8c** (89 ^2^)
**2**	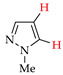 **2a**	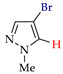 **2c** (76 ^3^)		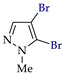 **2c’** (5 ^3^)	**9**	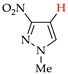 **9a**	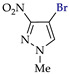 **9c** (15 ^3^)
**3**	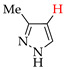 **3a**		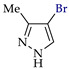 **3c** (66 ^3^)		**10**	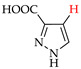 **10a**	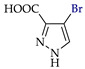 **10c** (68 ^3^)
**4**	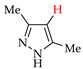 **4a**		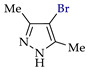 **4c** (94 ^2^)		**11**	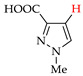 **11a**	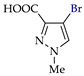 **11c** (78 ^2^)
**5**	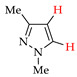 **5a**	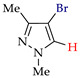 **5c** (55 ^3^)		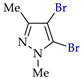 **5c’** (26 ^3^)	**12**	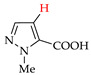 **12a**	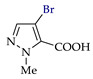 **12c** (84 ^2^)
**6**	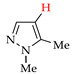 **6a**		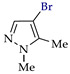 **6c** (88 ^2^)		**13**	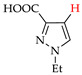 **13a**	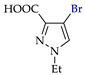 **13c** (84 ^2^)
**7**	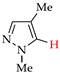 **7a**		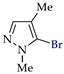 **7c** (0 ^5^)		**14**	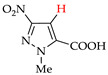 **14a**	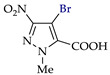 **14c** (0 ^3^)

^1^ Electrolysis in 100 mL of 1M solution of NaBr in H_2_O–CHCl_3_ (entries 1–7), H_2_O (entries 8–14), 30 °C, pyrazole (12.5–50 mmol), divided cell, Pt anode, Cu cathode, galvanostatic electrolysis (*j*_anode_ = 30 mA·cm^−2^), Q = Q_t_ = 2412–9650 C; ^2^ the yield was determined for the isolated product; ^3^ the yield was calculated from the ^1^H NMR spectroscopic data for the isolated mixture of products with (or) unreacted pyrazoles; ^5^ unpublished data.

**Table 3:** C‒H (An) iodination of pyrazoles (Az‒H).

Entry	Az‒H, Az‒I (Yield, %) [73,74]		Entry	Az‒H, Az‒I (Yield, %) [73,74]	
**1**	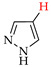 **1a**	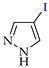 **1d** (57 ^1,3^, 93 ^2,3^)	**8**	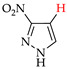 **8a**	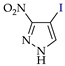 **8d** (2 ^1,4^, 82 ^2,3^)
**2**	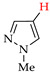 **2a**	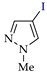 **2d** (5 ^1,4^, 79 ^2,3^)	**9**	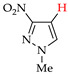 **9a**	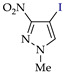 **9d** (0 ^1,4^)
**3**	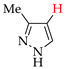 **3a**	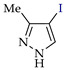 **3d** (71 ^1,3^)	**10**	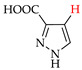 **10a**	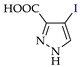 **10d** (30 ^1,4^, 86 ^2,3^)
**4**	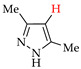 **4a**	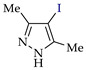 **4d** (86 ^1,3^, 93 ^2,3^)	**11**	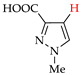 **11a**	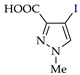 **11d** (0 ^1,4^, 74 ^2,3^)
**5**	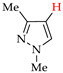 **5a**	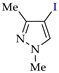 **5d** (35 ^1,4^)	**12**	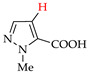 **12a**	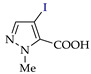 **12d** (0 ^1,4^, 78 ^2,3^)
**6**	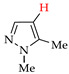 **6a**	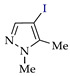 **6d** (42 ^1,5^)	**13**	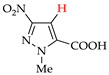 **14a**	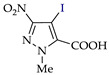 **14d** (0 ^1,5^, 79 ^2,3^)
**7**	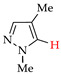 **7a**	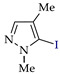 **7d** (0 ^1,5^)	**14**	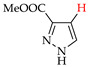 **15a**	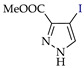 **15d** (40 ^1,4^)

^1^ Electrolysis in 100 mL of 0.3M solution of NaNO_3_ in H_2_O–CHCl_3_, 30 °C, KI (10 mmol), pyrazole (10 mmol), NaHCO_3_ (15 mmol), divided cell, Pt anode, Cu cathode, galvanostatic electrolysis (*j*_anode_ = 7.5 mA·cm^−2^), Q = Q_t_ = 1930 C; ^2^ two-step process: 1. electrogeneration of KIO_3_ in 1M aq. KOH, 70 °C, KI (30 mmol), K_2_Cr_2_O_7_ (0.7 mmol), undivided cell, NiO(OH) anode, Ni cathode, galvanostatic electrolysis (*j*_anode_ = 200 mA·cm^−2^), Q_t_ = 17370 C, Q/Q_t_ = 0.9–1.1; 2. pyrazole (45–150 mmol), KIO_3_ (9–30 mmol), I_2_ (18–60 mmol), H_2_O–CHCl_3_ (or H_2_O–CCl_4_), H_2_SO_4_ conc., temperature 50–66 °C, 0.5–14 h; ^3^ the yield was determined for the isolated product; ^4^ the yields were calculated from the ^1^H NMR spectroscopic data for the isolated products with (or) unreacted pyrazoles; ^5^ unpublished data.

**Table 4:** C–H An thiocyanation of pyrazole derivatives (**Az‒H**) via (SCN)_2_ (approach A) ^1^.

Entry	Az–H, Az–SCN (Yield, %) [22,122,123,126]		Entry	Az–H, Az–SCN (Yield, %) [22,122,123,126]	
**1**	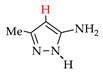 **1e**	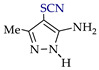 **1f** (83 ^2,5,6^, 72 ^2,4,6^, 74 ^2,5,7^, 69 ^3,5,7^)	**6**	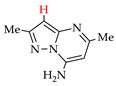 **6e**	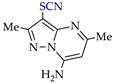 **6f** (86 ^2,4,6^)
**2**	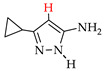 **2e**	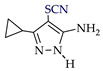 **2f** (87 ^2,5,6^, 78 ^2,5,7^, 71 ^3,5,7^)	**7**	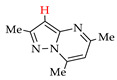 **7e**	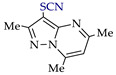 **7f** (83 ^2,4,6^, 80 ^2,4,7^, 75 ^2,5,7^, 77 ^3,4,7^, 71 ^3,5,7^)
**3**	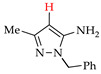 **3e**	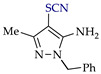 **3f** (65 ^2,4,6^, 57 ^3,4,6^)	**8**	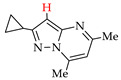 **8e**	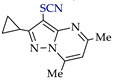 **8f** (85 ^2,4,6^, 82 ^2,5,6^)
**4**	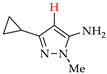 **4e**	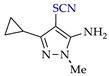 **4f** (75 ^2,4,6^, 68 ^3,4,6^)	**9**	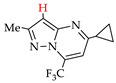 **9e**	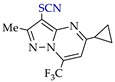 **9f** (75 ^2,4,6^, 66 ^2,5,6^)
**5**	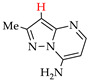 **5e**	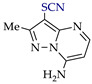 **5f** (89 ^2,4,6^, 64 ^2,5,6^)			

^1^ Electrolysis in 50–85 mL of 0.1M solution of NaClO_4_ in MeCN-H_2_O (entries 1–2), MeCN (entries 3–5, 7–9), MeCN–MeOH (entry 6), 20–25 °C, NH_4_SCN (3–20 mmol), pyrazole (entries 1–5, 7–9) or its hydrochloride (entry 6) (1–6 mmol), divided cell (entries 1–2), undivided cell (entries 3–9), Q_t_ = 193–985 C, Q/Q_t_ = 1 (entries 1–6), Q/Q_t_ = 2 (entries 7–9). All yields were determined for the isolated and purified products; ^2^ CPE—controlled potential electrolysis (E_anode_ = 0.70–1.00 V); ^3^ GE—galvanostatic electrolysis (*j*_anode_ = 2.50–12.50 mA·cm^−2^); ^4^ Pt electrodes; ^5^ GC electrodes; ^6^ milligram scale of electrosynthesis; ^7^ gram scale of electrosynthesis.

**Table 5:** C–H An thiocyanation of hardly oxidizable pyrazolo[1,5-a]pyrimidines (**Az‒H)** (approaches B and C) ^1^.

Entry	Az–H (E_p_^ox^, V^2^), Az–SCN (Yield, %) [122,123]		Entry	Az–H (E_p_^ox^, V^2^), Az–SCN (Yield, %) [122,123]	
**1**	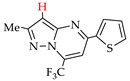 **10e** (1.75)	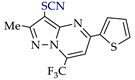 **10f** (81 ^3,5^, 65 ^4,5^, 60 ^4,6^)	**4**	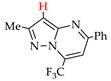 **13e** (1.85)	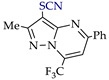 **13f** (80 ^3,5^, 62 ^4,5^, 55 ^4,6^)
**2**	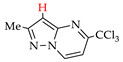 **11e** (1.77)	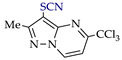 **11f** (79^3,5^, 60^4,5^)	**5**	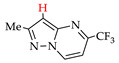 **14e** (1.85)	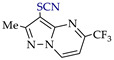 **14f** (73 ^3,5^, 60 ^4,5^)
**3**	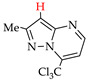 **12e** (1.79)	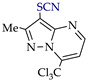 **12f** (77 ^3,5^, 60 ^4,5^, 52 ^4,6^)	**6**	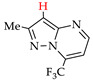 **15e** (1.88)	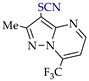 **15f** (69 ^3,5^, 63 ^4,5^, 47 ^4,6^)

^1^ Electrolysis in 60 mL 0.1 M solution of NaClO_4_ in MeCN, 20–25 °C, undivided cell, Q_t_ = 193 C. All yields was determined for the isolated and purified products; ^2^ it was determined by CV (working electrode—Pt, reference electrode—SCE, ν = 0.10 V·s^−1^); ^3^ Approach B: KSCN (4 mmol), ZnCl_2_ (2 mmol), pyrazole (1 mmol), CPE (E_anode_ = 1.00 V), Q_t_ =193 C, Q/Q_t_ = 3; ^4^ Approach C: NH_4_SCN (4 mmol), pyrazole (1 mmol), CPE (E_anode_ = 1.75–1.88 V), Q/Q_t_ = 3; ^5^ Pt electrodes; ^6^ GC electrodes.

**Table 6:** N–N homo-coupling of aminopyrazoles (Az–NH_2_) using approaches A, B, C (C’), D (D’) ^1^.

Entry	Az–NH_2_, Az–N=N–Az and Other Products (Yield, %) [161,163,165]		Entry	Az–NH_2_, Az–N=N–Az and Other Products (Yield, %) [161,163,165]	
**1**	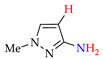 **1k**	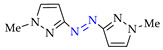 **1k-1k** (82 ^2^, 34 ^4^, 72 ^5^, 40 ^6^, 75 ^7^)	**7**	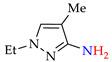 **5k**	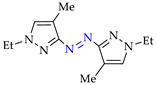 **5k-5k** (86 ^3^)
	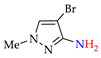 **1k′** (59^3^, 22^4^, 1^5^)	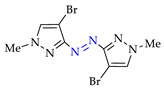 **1k′-1k′** (28^3^, 48 ^4^)	**8**	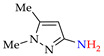 **6k**	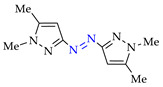 **6k-6k** (79^2^, 2 ^6^)
	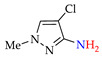 **1k″** (7^6^, 5^7^)	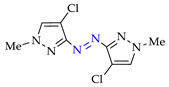 **1k″-1k″** (40 ^6^, 14 ^7^)		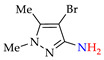 **6k′** (3 ^3^)	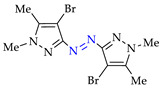 **6k′-6k′** (80 ^3^)
**2**	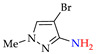 **1k′**	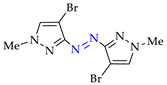 **1k′-1k′** (77 ^2^, 62 ^4^)		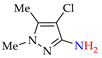 **6k″** (6)	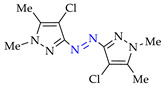 **6k″-6k″** (79 ^6^)
**3**	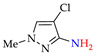 **1k″**	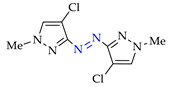 **1k″-1k″** (87 ^2^)	**9**	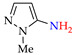 **7k**	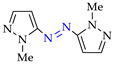 **7k-7k** (55 ^2^)
**4**	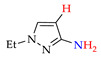 **2k**	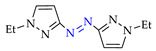 **2k-2k** (71^2^, 73^5^)	**10**	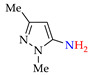 **8k**	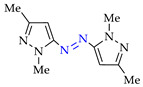 **8k-8k** (52 ^2^)
	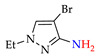 **2k′** (2^5^)	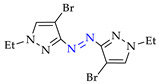 **2k′-2k′** (6 ^5^)	**11**	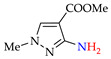 **9k**	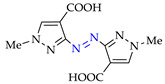 **9k-9k^8^** (67 ^2^)
**5**	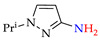 **3k**	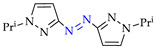 **3k-3k** (86 ^2^)	**12**	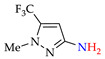 **10k**	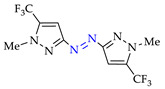 **10k-10k** (93 ^4^, 86 ^6^)
**6**	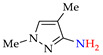 **4k**	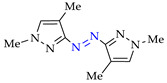 **4k-4k** (88 ^2^, 62 ^3^, 70 ^4^)			

^1^ Electrolysis in 100 mL of supporting electrolyte, 20–25 °C, undivided cell galvanostatic electrolysis; ^2^ Approach A: 0.5M aq. NaOH, NiO(OH) anode, pyrazole (3 mmol), *j*_anode_ = 6 mA·cm^−2^, Q_t_ = 579 C, Q/Q_t_ = 1–4; ^3^ Approach B: 2M aq. NaBr, RTOA, pyrazole (3 mmol), *j*_anode_ = 100 mA·cm^−2^, 48% HBr additives during electrolysis until reaching pH~7 (entries 1, 8), Q_t_ = 579 C, Q/Q_t_ = 1–4; ^4^ Approach C: 1. electrogeneration of NaOBr (HOBr) in 2M aq. NaBr, RTOA, *j*_anode_ = 100 mA·cm^−2^, Q = 661–1983 C; 2. pyrazole (2 mmol), NaOBr (HOBr) (2–4 mmol), 5 h; ^5^ Approach C’: see Approach C, but with NaOH (6 mmol) additives; ^6^ Approach D: 1. electrogeneration of HOCl (NaOCl) in 4M aq. NaCl, RTOA, *j*_anode_ = 161.5 mA·cm^−2^, Q= 588–1764 C; 2. Pyrazole (2 mmol), HOBr (NaOBr) (2–4 mmol), 5 h; ^7^ Approach D’: see Approach D, but with NaOH (6 mmol) additives; ^8^ it was identified after preparation and isolation of the corresponding methyl ester.

**Table 7:** N–N cross-coupling of aminopyrazoles (Az–NH_2_) using approach A ^1^.

Entry	Az^1^–NH_2_	H_2_N–Az^2^	Az^1^–N=N–Az^2^	Yield, %
**1**	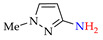 **1k**	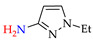 **2k**	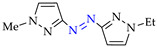 **1k-2k**	50 (**1k-2k**) 36 (**1k-1k**) 37(**2k-2k**)
**2**	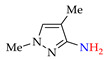 **4k**	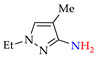 **5k**	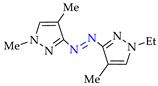 **4k-5k**	48 (**4k-5k**) 29 (**1k-1k**) 23 (**2k-2k**)

^1^ Electrolysis in 100 mL of 0.5 M aq. NaOH, 20–25 °C, undivided cell,; ^2^ Approach A: NiO(OH) anode, Ti cathode, pyrazole (1.5 mmol), galvanostatic electrolysis (*j*_anode_ = 6 mA·cm^−2^), Q = 2Q_t_ = 579 C.

## Sample Availability

Samples of the compounds **2b**, **4b**, **9b**–**13b**, **1c**–**4c**, **6c**, **8c**, **10c**–**13c**, **1d–4d**, **8d**, **10d–14d**, **1f**–**15f**, **1j**, **2j**, **1k-1k**–**8k-8k** are available from the authors.

## References

[1] Godula K., Sames D. (2006). C-H Bond Functionalization in Complex Organic Synthesis. Science.

[2] Davies H.M.L., Morton D. (2014). C-H Functionalization: Collaborative Methods to Redefine Chemical Logic. Angew. Chem. Int. Ed..

[3] Davies H.M.L., Morton D. (2016). Recent Advances in C–H Functionalization. J. Org. Chem..

[4] Smith M.B. (2020). Chapter 11. Aromatic Substitution, Electrophilic. March’s Advanced Organic Chemistry: Reactions, Mechanisms, and Structure.

[5] Chupakhin O.N., Postovskii I.Y. (1976). Nucleophilic Substitution of Hydrogen in Aromatic Systems. Russ. Chem. Rev..

[6] Chupakhin O.N., Charushin V.N., van der Plas H.C. (1994). Nucleophilic Aromatic Substitution of Hydrogen.

[7] Charushin V.N., Chupakhin O.N. (2007). Nucleophilic aromatic substitution of hydrogen and related reactions. Mendeleev Commun..

[8] Chupakhin O.N., Charushin V.N. (2016). Recent advances in the field of nucleophilic aromatic substitution of hydrogen. Tetrahedron Lett..

[9] Charushin V.N., Chupakhin O.N. (2019). Nucleophilic C—H functionalization of arenes: A contribution to green chemistry. Russ. Chem. Bull..

[10] Seebach D. (1979). Methods of Reactivity Umpolung. Angew. Chem. Int. Ed..

[11] Weinberg N.L., Weinberg H.R. (1968). Electrochemical oxidation of organic compounds. Chem. Rev..

[12] Baizer M.M. (1973). Organic Electrochemistry.

[13] Baizer M.M. (1983). Organic Electrochemistry.

[14] Yoshida K. (1984). Electrooxidation in Organic Chemistry. The Role of Cation Radicals as Synthetic Intermediates.

[15] Lund H., Baizer M.M. (1991). Organic Electrochemistry.

[16] Hammerich O., Utley J.H.P., Eberson L., Lund H., Hammerich O. (2001). Organic Electrochemistry.

[17] Shchepochkin A.V., Chupakhin O.N., Charushin V.N., Petrosyan V.A. (2013). Direct nucleophilic functionalization of C(sp^2^)–H-bonds in arenes and hetarenes by electrochemical methods. Russ. Chem. Rev..

[18] Francke R., Little R.D. (2014). Redox catalysis in organic electrosynthesis: Basic principles and recent developments. Chem. Soc. Rev..

[19] Karkas M.D. (2018). Electrochemical strategies for C-H functionalization and C-N bond formation. Chem. Soc. Rev..

[20] Yang Q.-L., Fang P., Mei T.-S. (2018). Recent Advances in Organic Electrochemical C–H Functionalization. Chin. J. Chem..

[21] Petrosyan V.A. (2011). Reactions of anodic and chemical aromatic substitution. Mendeleev Commun..

[22] Kokorekin V.A., Sigacheva V.L., Petrosyan V.A. (2014). New data on heteroarene thiocyanation by anodic oxidation of NH_4_SCN. The processes of electroinduced nucleophilic aromatic substitution of hydrogen. Tetrahedron Lett..

[23] Sheldon R.A., Arends I., Hanefeld U. (2007). Green Chemistry and Catalysis.

[24] Frontana-Uribe B.A., Little R.D., Ibanez J.G., Palma A., Vasquez-Medrano R. (2010). Organic electrosynthesis: A promising green methodology in organic chemistry. Green Chem..

[25] Heard D.M., Lennox A.J.J. (2020). Electrode Materials in Modern Organic Electrochemistry. Angew. Chem. Int. Ed..

[26] Lyalin B.V., Petrosyan V.A. (2013). Electrochemical halogenation of organic compounds. Russ. J. Electrochem..

[27] Beer H.B., Landau U., Yeager E., Kortan D. (1982). Dimensionally Stable Anodes. Electrochemistry in Industry: New Directions.

[28] Janin Y.L. (2012). Preparation and Chemistry of 3/5-Halogenopyrazoles. Chem. Rev..

[29] Slomczynska U., Olivo P., Beattie J., Starkey G., Noueiry A., Roth R. (2010). Compounds, Compositions and Methods for Control of Hepatitis C Viral Infections. International Patent.

[30] Tanigchi T., Kawada A., Kondo M., Quinn J.F., Kanitomo J., Yoshikawa M., Fashimi M. (2010). Pyridazinone Compounds. U.S. Patent.

[31] Alberati D., Alvares R.S., Bleicher K., Flohr A., Markus R., Groeke K.Z., Koerner M., Kuhn B., Peters J.-U., Rudolph M. (2011). Novel Imidazopyridines. U.S. Patent.

[32] Linn D.M., Fong E.H. (2004). Nicotinic Acetylcholine Agonists in the Treatment of Glaucoma and Retinal Neuropathy. International Patent.

[33] Tomokazu N. (2006). Therapeutic Agent for Hyperlipemia, Arteriosclerosis, and/or Metabolic Syndrome. Japan Patent.

[34] D’orchumont H., Fraisse L., Zimmerman A. (2005). Use of Indazolecarboxamide Derivatives for the Preparation of a Medicament that is Intended for the Treatment and Prevention of Paludism. International Patent.

[35] Pennell A.M.K., Aggen J.B., Wright J.J., Sen S., McMaster B.E., Brian E., Dairaghi J.D. (2003). 1-Aryl-4-substituted Piperazines Derivatives for Use as CCR1 Antagonists for the Treatment of Inflammation and Immune Disorders. International Patent.

[36] Singh J., Gurney M.E., Burgin A., Sandanayaka V., Kiselyov A., Motta A., Schultz G., Hategan G. (2009). Biaryl as PDE4 inhibitors for treating inflammation. International Patent.

[37] Talley J.J., Sprott K., Pearson J.P., Game P., Milene G.T., Schairer W., Yang J.J., Kim C., Barden T., Lundigran R. (2008). Indole compounds. International Patent.

[38] Pelcman B., Sanin A., Nilsson P., Groth T., Kromann H. (2007). Pyrazoles Useful in the Treatment of Inflammation. International Patent.

[39] Banner D., Helpert H., Mauser H., Mayweg V.A., Rogers-Evans M. (2011). Amino oxazine Derivatives. International Patent.

[40] Bamba M., Eiki J., Mitsuya M., Nishimura T., Sakai F., Sasaki Y., Watanabe H. (2004). Novel 2-Pyridinecarboxamide Derivatives. International Patent.

[41] Fix-Stenzel S., Judd A., Michaelides M. (2011). Pyrrolopyridine and Pyrrolopyrimidine Inhibitors of Kinases. International Patent.

[42] You H., Youn H.-S., Im I., Bae M.-H., Lee S.-K., Ko H., Eom S.H., Kim Y.-C. (2011). Design, synthesis and X-ray crystallographic study of NAmPRTase inhibitors as anti-cancer agents. Eur. J. Med. Chem..

[43] Ahearn S.P., Altman M., Chichetti S., Czako B., Daniels M.H., Falcone D., Guerin D., Lipford K., Martinez M., Osimboni E. (2011). Tyrosine kinase Inhibitors. International Patent.

[44] Basso A.D. (2009). Anti-mitotic Agent and Aurora Kinase Inhibitor Combination as Anti-cancer Treatment. International Patent.

[45] Hattori K., Matsuda K., Murata M., Nakajima T., Tsutsumi H. (1993). Substituted 3-Pyrrolidinylthio-carbapenems as Antimicrobial Agents. International Patent.

[46] Barrett D., Matsuda H., Matsuda K., Matsuya T., Mizuno H., Murano K., Ogino T., Toda A. (2002). New Compound. International Patent.

[47] Kudo N., Furuta S., Taniguchi M., Endo T., Sato K. (1999). Synthesis and Herbicidal Activity of 1,5-Diarylpyrazole Derivatives. Chem. Pharm. Bull..

[48] Fustero S., Román R., Sanz-Cervera J.F., Simón-Fuentes A., Cuñat A.C., Villanova S., Murguía M. (2008). Improved Regioselectivity in Pyrazole Formation through the Use of Fluorinated Alcohols as Solvents: Synthesis and Biological Activity of Fluorinated Tebufenpyrad Analogs. J. Org. Chem..

[49] Higashino Y., Ikeda Y., Koike S., Kyomura N., Okui S., Tomita H. (1997). Pyrazoles and Agricultural Chemicals Containing Them as Active Ingredients. International Patent.

[50] Auler T., Bojack G., Bretschneider T., Drewes M.W., Feucht D., Fischer R., Hills M., Kehne H., Konze J., Kuck K.-H. (2004). N-Heterocyclyl Phenyl-substituted Cyclic Ketoenols. International Patent.

[51] Fischer R., Gomibuchi T., Nakakura N., Otsu Y., Shibuya K., Wada K., Yoneta Y. (2005). N1-((Pyrazol-1-ymethyl)-2-methylphenyl)-phatalamide Derivatives and Related Compounds Insecticides. International Patent.

[52] Hüttel R., Schäfer O., Welzel G. (1956). Die Chlorierung der Pyrazole. Justus Liebigs Ann. Chem..

[53] Ohsawa A., Kaihoh T., Itoh T., Okada M., Kawabata C., Yamaguchi K., Igeta H. (1988). Reactions of N-Aminopyrazoles with Halogenating Reagents and Synthesis of 1, 2, 3-Triazines. Chem. Pharm. Bull..

[54] Stefani H.A., Pereira C.M.P., Almeida R.B., Braga R.C., Guzen K.P., Cella R. (2005). A mild and efficient method for halogenation of 3,5-dimethyl pyrazoles by ultrasound irradiation using N-halosuccinimides. Tetrahedron Lett..

[55] Zhao Z.G., Wang Z.X. (2007). Halogenation of Pyrazoles Using N-Halosuccinimides in CCl_4_ and in Water. Synthetic Commun..

[56] Mullens P.R. (2009). An improved synthesis of 1-methyl-1H-pyrazole-4-boronic acid pinacol ester and its corresponding lithium hydroxy ate complex: Application in Suzuki couplings. Tetrahedron Lett..

[57] Khan K.M., Maharvi G.M., Choudhary M.I., Rahman A.-U., Perveen S. (2005). A modified, economical and efficient synthesis of variably substituted pyrazolo[4,3-d]pyrimidin-7-ones. J. Het. Chem..

[58] Kerr E.R., Mather W.B. (1972). Electrochemical Chlorination of Hydrocarbon in Hydrochloric Acid-Acetic Acid Solytion. U.S. Patent.

[59] Ehlert M.K., Storr A., Thompson R.C. (1992). Metal pyrazolate polymers. Part 3. Synthesis and study of Cu(I) and Cu(II) complexes of 4-Xdmpz (where X = H, Cl, Br, I, and CH_3_ for Cu(I) and X = H, Cl, Br, and CH_3_ for Cu(II); dmpz = 3,5-dimethylpyrazolate). Can. J. Chem..

[60] Petko K., Sokolenko T., Yagupolskii L. (2006). Chemical properties of derivatives of N-difluoromethyl-and N-2-H-tetrafluoroethylpyrazoles. Chem. Heterocycl. Compd..

[61] Akopyan G.A. (2007). Bromination of pyrazole-3(5)-carboxylic acid. Russ. J. Gen. Chem..

[62] Guillou S., Bonhomme F.J., Ermolenko M.S., Janin Y.L. (2011). Simple preparations of 4 and 5-iodinated pyrazoles as useful building blocks. Tetrahedron.

[63] Salanouve E., Guillou S., Bizouarne M., Bonhomme F.J., Janin Y.L. (2012). 3-Methoxypyrazoles from 1,1-dimethoxyethene, few original results. Tetrahedron.

[64] Guillou S., Janin Y.L. (2010). 5-Iodo-3-Ethoxypyrazoles: An Entry Point to New Chemical Entities. Chem. Eur. J..

[65] Sinyakov A., Vasilevskii S., Shvartsberg M. (1977). A new rearrangement of chloroethynylpyrazoles. Russ. Chem. Bull..

[66] Vasilevskii S.F., Shvartsberg M.S. (1980). Oxidative iodination of substituted N-methylpyrazoles. Russ. Chem. Bull..

[67] Tret’yakov E.V., Vasitevsky S.F. (1996). Nitrodeiodination of 4-iodo-l-methylpyrazoles. Russ. Chem. Bull..

[68] Lyalin B.V., Petrosyan V.A., Ugrak B.I. (2009). Electrosynthesis of 4-chloropyrazolecarboxylic acids. Russ. Chem. Bull..

[69] Perevalov V.P., Manaev Y.A., Bezborodov B.V., Stepanov B.I. (1990). Chlorination of 1,5-dimethylpyrazole. Chem. Heterocycl. Compd..

[70] Lyalin B.V., Petrosyan V.A., Ugrak B.I. (2008). Electrosynthesis of 4-chloro derivatives of pyrazole and alkylpyrazoles. Russ. J. Electrochem..

[71] Lyalin B.V., Petrosyan V.A. (2014). The Reactivity Trends in Electrochemical Chlorination and Bromination of N-Substituted and N-Unsubstituted Pyrazoles. Chem. Heterocycl. Compd..

[72] Lyalin B.V., Petrosyan V.A., Ugrak B.I. (2010). Electrosynthesis of 4-bromosubstituted pyrazole and its derivatives. Russ. J. Electrochem..

[73] Lyalin B.V., Petrosyan V.A., Ugrak B.I. (2010). Electrosynthesis of 4-iodopyrazole and its derivatives. Russ. Chem. Bull..

[74] Lyalin B.V., Petrosyan V.A. (2014). New approach to electrochemical iodination of arenes exemplified by the synthesis of 4-iodopyrazoles of different structures. Russ. Chem. Bull..

[75] Lyalin B.V., Petrosyan V.A. (2013). Efficient iodination of structurally varying pyrazoles in heterophase medium. Russ. Chem. Bull..

[76] Noszticzius Z., Noszticzius E., Schelly Z.A. (1982). Use of ion-selective electrodes for monitoring oscillating reactions. 1. Potential response of the silver halide membrane electrodes to hypohalous acids. J. Am. Chem. Soc..

[77] Kanishchev M.I., Korneeva N.V., Shevelev S.A., Fainzil’berg A.A. (1988). Nitropyrazoles (review). Chem. Heterocycl. Compd..

[78] Belen’kii L.I., Chuvylkin N.D. (1996). Relationships and features of electrophilic substitution reactions in the azole series. Chem. Heterocycl. Compd..

[79] Pevzner M.S., Katritzky A.R. (1999). Aromatic N-Haloazoles. Advances in Heterocyclic Chemistry.

[80] Lyalin B.V., Petrosyan V.A. (2010). Oxidation of organic compounds on NiOOH electrode. Russ. J. Electrochem..

[81] Nikoofar K. (2013). A Brief on Thiocyanation of N-Activated Arenes and N-Bearing Heteroaromatic Compounds. Chem. Sci. Trans..

[82] Castanheiro T., Suffert J., Donnard M., Gulea M. (2016). Recent advances in the chemistry of organic thiocyanates. Chem. Soc. Rev..

[83] Majedi S., Sreerama L., Vessally E., Behmagham F. (2020). Metal-Free Regioselective Thiocyanation of (Hetero) Aromatic C-H Bonds using Ammonium Thiocyanate: An Overview. J. Chem. Lett..

[84] Rezayati S., Ramazani A. (2020). A review on electrophilic thiocyanation of aromatic and heteroaromatic compounds. Tetrahedron.

[85] Maurya C.K., Mazumder A., Gupta P.K. (2017). Phosphorus pentasulfide mediated conversion of organic thiocyanates to thiols. Beilstein J. Org. Chem..

[86] Noland W.E., Lanzatella N.P., Dickson R.R., Messner M.E., Nguyen H.H. (2013). Access to Indoles via Diels–Alder Reactions of 5-Methylthio-2-vinylpyrroles with Maleimides. J. Heterocycl. Chem..

[87] Maurya C.K., Mazumder A., Kumar A., Gupta P.K. (2016). Synthesis of Disulfanes from Organic Thiocyanates Mediated by Sodium in Silica Gel. Synlett.

[88] Biswas K., Ghosh S., Ghosh P., Basu B. (2016). Cyclic ammonium salts of dithiocarbamic acid: Stable alternative reagents for the synthesis of S-alkyl carbodithioates from organyl thiocyanates in water. J. Sulfur Chem..

[89] Li W.-T., Wu W.-H., Tang C.-H., Tai R., Chen S.-T. (2011). One-Pot Tandem Copper-Catalyzed Library Synthesis of 1-Thiazolyl-1,2,3-triazoles as Anticancer Agents. ACS Comb. Sci..

[90] Baghershiroudi M., Safa K.D., Adibkia K., Lotfipour F. (2018). Synthesis and antibacterial evaluation of new sulfanyltetrazole derivatives bearing piperidine dithiocarbamate moiety. Synth. Commun..

[91] Kokorekin V.A., Terent’ev A.O., Ramenskaya G.V., Grammatikova N.É., Rodionova G.M., Ilovaiskii A.I. (2013). Synthesis and Antifungal Activity of Arylthiocyanates. Pharm. Chem. J..

[92] Fortes M.P., da Silva P.B.N., da Silva T.G., Kaufman T.S., Militão G.C.G., Silveira C.C. (2016). Synthesis and preliminary evaluation of 3-thiocyanato-1H-indoles as potential anticancer agents. Eur. J. Med. Chem..

[93] Chao M.N., Matiuzzi C.E., Storey M., Li C., Szajnman S.H., Docampo R., Moreno S.N.J., Rodriguez J.B. (2015). Aryloxyethyl Thiocyanates Are Potent Growth Inhibitors of Trypanosoma cruzi and Toxoplasma gondii. ChemMedChem.

[94] Kokorekin V.A., Khodonov V.M., Neverov S.V., Grammatikova N.É., Petrosyan V.A. (2021). “Metal-free” synthesis and antifungal activity of 3-thiocyanatopyrazolo[1,5-a]pyrimidines. Russ. Chem. Bull..

[95] Yi X.-J., El-Idreesy T.T., Eldebss T.M.A., Farag A.M., Abdulla M.M., Hassan S.A., Mabkhot Y.N. (2016). Synthesis, biological evaluation, and molecular docking studies of new pyrazol-3-one derivatives with aromatase inhibition activities. Chem. Biol. Drug Des..

[96] Wood J.L. (1946). Substitution and Addition Reactions of Thiocyanogen. Organic Reactions.

[97] Guy R.G., Patai S. (1977). Syntheses and preparative applications of thiocyanates. Cyanates and Their Thio Derivatives.

[98] Jason A., Phadke A.S., Deshpande M., Agarwak A., Chen D., Gadhachanda V.R., Hashimoto A., Pais G., Wang Q., Wang X. (2017). Aryl, Heteroaryl, and Heterocyclic Compounds for Treatment of Medical Disorders.

[99] Rodríguez R., Camargo P., Sierra C.A., Soto C.Y., Cobo J., Nogueras M. (2011). Iodine mediated an efficient and greener thiocyanation of aminopyrimidines by a modification of the Kaufmann’s reaction. Tetrahedron Lett..

[100] Reddy B.V.S., Reddy S.M.S., Madan C. (2011). NBS or DEAD as effective reagents in α-thiocyanation of enolizable ketones with ammonium thiocyanate. Tetr. Lett..

[101] Mahajan U.S., Akamanchi K.G. (2009). Facile Method for Thiocyanation of Activated Arenes Using Iodic Acid in Combination with Ammonium Thiocyanate. Synthetic Commun..

[102] Khazaei A., Zolfigol M.A., Mokhlesi M., Panah F.D., Sajjadifar S. (2012). Simple and Highly Efficient Catalytic Thiocyanation of Aromatic Compounds in Aqueous Media. Helv. Chim. Acta.

[103] Wu J., Wu G., Wu L. (2008). Thiocyanation of Aromatic and Heteroaromatic Compounds using Ammonium Thiocyanate and I_2_O_5_. Synthetic Commun..

[104] Ali D., Panday A.K., Choudhury L.H. (2020). Hydrogen Peroxide-Mediated Rapid Room Temperature Metal-Free C(sp2)-H Thiocyanation of Amino Pyrazoles, Amino Uracils, and Enamines. J. Org. Chem..

[105] Sajjadifar S., Louie O. (2013). Regioselective Thiocyanation of Aromatic and Heteroaromatic Compounds by Using Boron Sulfonic Acid as a New, Efficient, and Cheap Catalyst in Water. J. Chem..

[106] Khalili D. (2015). Highly efficient and regioselective thiocyanation of aromatic amines, anisols and activated phenols with H_2_O_2_/NH_4_SCN catalyzed by nanomagnetic Fe_3_O_4_. Chin. Chem. Lett..

[107] Khazaei A., Zolfigol M.A., Mokhlesi M., Pirveysian M. (2012). Citric acid as a trifunctional organocatalyst for thiocyanation of aromatic and heteroaromatic compounds in aqueous media. Can. J. Chem..

[108] Songsichan T., Katrun P., Khaikate O., Soorukram D., Pohmakotr M., Reutrakul V., Kuhakarn C. (2018). Thiocyanation of Pyrazoles Using KSCN/K_2_S_2_O_8_ Combination. SynOpen.

[109] Mete T.B., Khopade T.M., Bhat R.G. (2017). Transition-metal-free regioselective thiocyanation of phenols, anilines and heterocycles. Tetrahedron Lett..

[110] Karimi Zarchi M.A., Banihashemi R. (2016). Thiocyanation of aromatic and heteroaromatic compounds using polymer-supported thiocyanate ion as the versatile reagent and ceric ammonium nitrate as the versatile single-electron oxidant. J. Sulfur Chem..

[111] Pan X.-Q., Lei M.-Y., Zou J.-P., Zhang W. (2009). Mn(OAc)_3_-promoted regioselective free radical thiocyanation of indoles and anilines. Tetrahedron Lett..

[112] Das B., Kumar A.S. (2010). Efficient Thiocyanation of Indoles Using Para-Toluene Sulfonic Acid. Synth. Commun..

[113] Zhang H., Wei Q., Wei S., Qu J., Wang B. (2016). Highly Efficient and Practical Thiocyanation of Imidazopyridines Using an N-Chlorosuccinimide/NaSCN Combination. Eur. J. Org. Chem..

[114] Muniraj N., Dhineshkumar J., Prabhu K.R. (2016). N-Iodosuccinimide Catalyzed Oxidative Selenocyanation and Thiocyanation of Electron Rich Arenes. ChemistrySelect.

[115] Mokhtari B., Azadi R., Rahmani-Nezhad S. (2009). In situ-generated N-thiocyanatosuccinimide (NTS) as a highly efficient reagent for the one-pot thiocyanation or isothiocyanation of alcohols. Tetrahedron Lett..

[116] Memarian H.R., Mohammadpoor-Baltork I., Nikoofar K. (2007). DDQ-promoted thiocyanation of aromatic and heteroaromatic compounds. Can. J. Chem..

[117] Memarian H.R., Mohammadpoor-Baltork I., Nikoofar K. (2008). Ultrasound-assisted thiocyanation of aromatic and heteroaromatic compounds using ammonium thiocyanate and DDQ. Ultrason. Sonochem..

[118] Gitkis A., Becker J.Y. (2010). Anodic thiocyanation of mono- and disubstituted aromatic compounds. Electrochim. Acta.

[119] Fotouhi L., Nikoofar K. (2013). Electrochemical thiocyanation of nitrogen-containing aromatic and heteroaromatic compounds. Tetrahedron Lett..

[120] Zhang X., Wang C., Jiang H., Sun L. (2018). A low-cost electrochemical thio- and selenocyanation strategy for electron-rich arenes under catalyst- and oxidant-free conditions. RSC Adv..

[121] Kokorekin V.A., Yaubasarova R.R., Neverov S.V., Petrosyan V.A. (2016). Reactivity of electrogenerated thiocyanogen in the thiocyanation of pyrazolo[1,5-a]pyrimidines. Mendeleev Commun..

[122] Kokorekin V.A., Mel’nikova E.I., Yaubasarova R.R., Gorpinchenko N.V., Petrosyan V.A. (2019). “Metal-free” electrooxidative C—H thiocyanation of arenes. Russ. Chem. Bull..

[123] Kokorekin V.A., Yaubasarova R.R., Neverov S.V., Petrosyan V.A. (2019). Electrooxidative C–H Functionalization of Heteroarenes. Thiocyanation of Pyrazolo[1,5-a]pyrimidines. Eur. J. Org. Chem..

[124] Yaubasarova R.R., Kokorekin V.A., Ramenskaya G.V., Petrosyan V.A. (2019). Double electrooxidative C–H functionalization of (het)arenes with thiocyanate and 4-nitropyrazolate ions. Mendeleev Commun..

[125] Kokorekin V.A., Melnikova E.I., Yaubasarova R.R., Petrosyan V.A. (2020). Electrooxidative C–H thiocyanation of hetarenes: Voltammetric assessment of thiocyanogen reactivity. Mendeleev Commun..

[126] Kokorekin V.A., Neverov S.V., Kuzina V.N., Petrosyan V.A. (2020). A New Method for the Synthesis of 3-Thiocyanatopyrazolo[1,5-a]pyrimidines. Molecules.

[127] Cataldo F. (1997). New Developments in the Study of the Structure of Parathiocyanogen: (SCN)_x_, An Inorganic Polymer. J. Inorg. Organomet. Polym..

[128] Shaabani A., Nazeri M.T., Afshari R. (2019). 5-Amino-pyrazoles: Potent reagents in organic and medicinal synthesis. Mol. Divers..

[129] Cherukupalli S., Karpoormath R., Chandrasekaran B., Hampannavar G.A., Thapliyal N., Palakollu V.N. (2017). An insight on synthetic and medicinal aspects of pyrazolo[1,5-a]pyrimidine scaffold. Eur. J. Med. Chem..

[130] Arias-Gómez A., Godoy A., Portilla J. (2021). Functional Pyrazolo[1,5-a]pyrimidines: Current Approaches in Synthetic Transformations and Uses as an Antitumor Scaffold. Molecules.

[131] Sigacheva V.L., Kokorekin V.A., Strelenko Y.A., Neverov S.V., Petrosyan V.A. (2012). Electrochemical Azolation of N-substituted Pyrroles: A New Case in S_N_^H^(An) Reactions. Mendeleev Commun..

[132] Henton D.R., McCreery R.A., Swenton J.S. (1980). Anodic oxidation of 1,4-dimethoxy aromatic compounds. A facile route to functionalized quinone bisketals. J. Org. Chem..

[133] Adibi H., Rashidi A., Khodaei M.M., Alizadeh A., Majnooni M.B., Pakravan N., Abiri R., Nematollahi D. (2011). Catecholthioether Derivatives: Preliminary Study of in-Vitro Antimicrobial and Antioxidant Activities. Chem. Pharm. Bull..

[134] Kokorekin V.A., Solomatin Y.A., Gening M.L., Petrosyan V.A. (2017). Acid-catalyzed S_N_^H^(An) thiolation of p-dihydroxybenzene. Mendeleev Commun..

[135] Chen C., Niu P., Shen Z., Li M. (2018). Electrochemical Sulfenylation of Indoles with Disulfides Mediated by Potassium Iodide. J. Electrochem. Soc..

[136] Petrosyan V.A., Burasov A.V. (2010). Anodic azolation of 1,2- and 1,3-dimethoxybenzenes. Russ. Chem. Bull..

[137] Feng P., Ma G., Chen X., Wu X., Lin L., Liu P., Chen T. (2019). Electrooxidative and Regioselective C−H Azolation of Phenol and Aniline Derivatives. Angew. Chem. Int. Ed..

[138] Wang J.-H., Lei T., Nan X.-L., Wu H.-L., Li X.-B., Chen B., Tung C.-H., Wu L.-Z. (2019). Regioselective Ortho Amination of an Aromatic C–H Bond by Trifluoroacetic Acid via Electrochemistry. Org. Lett..

[139] Ananikov V.P., Eremin D.B., Yakukhnov S.A., Dilman A.D., Levin V.V., Egorov M.P., Karlov S.S., Kustov L.M., Tarasov A.L., Greish A.A. (2017). Organic and hybrid systems: From science to practice. Mendeleev Commun..

[140] Hunger K. (2003). Industrial Dyes: Chemistry, Properties, Applications.

[141] Sandborn W.J. (2002). Rational selection of oral 5-aminosalicylate formulations and prodrugs for the treatment of ulcerative colitis. Am. J. Gastroenterol..

[142] Ovchinnikov I.V., Kulikov A.S., Makhova N.N., Tosco P., Di Stilo A., Fruttero R., Gasco A. (2003). Synthesis and vasodilating properties of N-alkylamide derivatives of 4-amino-3-furoxancarboxylic acid and related azo derivatives. Il Farmaco.

[143] Iranpoor N., Firouzabadi H., Khalili D., Motevalli S. (2008). Easily Prepared Azopyridines as Potent and Recyclable Reagents for Facile Esterification Reactions. An Efficient Modified Mitsunobu Reaction. J. Org. Chem..

[144] Iranpoor N., Firouzabadi H., Shahin R., Khalili D. (2011). 2,2′-Azobenzothiazole as a New Recyclable Oxidant for Heterogeneous Thiocyanation of Aromatic Compounds with Ammonium Thiocyanate. Synth. Commun..

[145] Ovchinnikov I.V., Makhova N.N., Khmel’nitsky L.I., Kuz’min V.S., Akimova L.N., Pepekin V.I. (1998). Dinitrodiazenofuroxan as a new energetic explosive. Dokl. Chem..

[146] Veauthier J.M., Chavez D.E., Tappan B.C., Parrish D.A. (2010). Synthesis and Characterization of Furazan Energetics ADAAF and DOATF. J. Energ. Mater..

[147] Firouzabadi H., Mostafavipoor Z. (1983). Barium Manganate. A Versatile Oxidant in Organic Synthesis. Bull. Chem. Soc. Jpn..

[148] Birchall J.M., Haszeldine R.N., Kemp J.E.G. (1970). Polyfluoroarenes. Part X. Polyfluoroaromatic azo-compounds. J. Chem. Soc. C.

[149] Farhadi S., Zaringhadama P., Sahamiehb R.Z. (2007). Photo-assisted Oxidation of Anilines and Other Primary Aromatic Amines to Azo Compounds Using Mercury (II) Oxide as a Photo-Oxidant. Acta Chim. Slov..

[150] Huang H., Sommerfeld D., Dunn B.C., Lloyd C.R., Eyring E.M. (2001). Ferrate(VI) oxidation of aniline. J. Chem. Soc. Dalton Trans..

[151] Chavez D.E., Parrish D.A., Leonard P. (2012). The Synthesis and Characterization of a New Furazan Heterocyclic System. Synlett.

[152] Okumura S., Lin C.-H., Takeda Y., Minakata S. (2013). Oxidative Dimerization of (Hetero)aromatic Amines Utilizing t-BuOI Leading to (Hetero)aromatic Azo Compounds: Scope and Mechanistic Studies. J. Org. Chem..

[153] Zhang C., Jiao N. (2010). Copper-Catalyzed Aerobic Oxidative Dehydrogenative Coupling of Anilines Leading to Aromatic Azo Compounds using Dioxygen as an Oxidant. Angew. Chem..

[154] Jiang B., Ning Y., Fan W., Tu S.-J., Li G. (2014). Oxidative Dehydrogenative Couplings of Pyrazol-5-amines Selectively Forming Azopyrroles. J. Org. Chem..

[155] Takeda Y., Okumura S., Minakata S. (2012). Oxidative Dimerization of Aromatic Amines using tBuOI: Entry to Unsymmetric Aromatic Azo Compounds. Angew. Chem. Int. Ed..

[156] Pandit R.P., Lee Y.R. (2015). Construction of Multifunctionalized Azopyrazoles by Silver- Catalyzed Cascade Reaction of Diazo Compounds. Adv. Synth. Catal..

[157] Wawzonek S., McIntyre T. (1967). Electrolytic oxidation of aromatic amines. J. Electrochem. Soc..

[158] Wawzonek S., McIntyre T. (1972). Electrolytic preparation of azobenzenes. J. Electrochem. Soc..

[159] Schäfer H.-J., Steckhan E. (1987). Oxidation of organic compounds at the nickel hydroxide electrode. Electrochemistry I. Top. Curr. Chem.

[160] Lyalin B.V., Sigacheva V.L., Kokorekin V.A., Petrosyan V.A. (2015). A new synthesis of azopyrazoles by oxidation of C-aminopyrazoles on a NiO(OH) electrode. Mendeleev Commun..

[161] Lyalin B.V., Sigacheva V.L., Kokorekin V.A., Petrosyan V.A. (2018). Electrosynthesis of azopyrazoles via the oxidation of N-alkylaminopyrazoles on a NiO(OH) anode in aqueous alkali—A green method for N-N homocoupling. Tetrahedron Lett..

[162] Lyalin B.V., Sigacheva V.L., Petrosyan V.A. (2020). Electrocatalytic N—N cross-coupling of N-alkyl-3-aminopyrazoles at a nickel anode. Russ. Chem. Bull..

[163] Lyalin B.V., Sigacheva V.L., Kokorekin V.A., Dutova T.Y., Rodionova G.M., Petrosyan V.A. (2018). Oxidative transformation of N-substituted 3-aminopyrazoles to azopyrazoles using electrogenerated bromine as a mediator. Russ. Chem. Bull..

[164] Lyalin B.V., Sigacheva V.L., Kokorekin V.A., Petrosyan V.A. (2017). Oxidative conversion of N-substituted 3-aminopyrazoles to azopyrazoles using electrogenerated NaOCl as the mediator. Arkivoc.

[165] Lyalin B.V., Sigacheva V.L., Ugrak B.I., Petrosyan V.A. (2021). Oxidative N—N coupling of N-alkyl-3-aminopyrazoles to azopyrazoles in aqueous solutions of NaOCl and NaOBr. Russ. Chem. Bull..

